# Severe clinical manifestation of mitochondrial 3-hydroxy-3-methylglutaryl-CoA synthase deficiency associated with two novel mutations: a case report

**DOI:** 10.1186/s12887-019-1747-5

**Published:** 2019-10-09

**Authors:** Hao Liu, Jing-kun Miao, Chao-wen Yu, Ke-xing Wan, Juan Zhang, Zhao-jian Yuan, Jing Yang, Dong-juan Wang, Yan Zeng, Lin Zou

**Affiliations:** 1 Center for Clinical Molecular Medicine, Chongqing, 400014 People’s Republic of China; 2 Ministry of Education Key Laboratory of Child Development and Disorder, Chongqing, 400014 People’s Republic of China; 3 National Clinical Research Center for Child Health and Disorders, Chongqing, 400014 People’s Republic of China; 4 China International Science and Technology Cooperation base of Child Development and critical Disorders, Chongqing, 400014 People’s Republic of China; 5 Chongqing key Laboratory of Pediatrics, Chongqing, 400014 People’s Republic of China; 6 Children’s Hospital of Chongqing Medical University, Chongqing, 400014 People’s Republic of China; 7 Department of Endocrinology, Chongqing, 400014 People’s Republic of China

**Keywords:** Inherited metabolic disorders, Mitochondrial 3-hydroxy-3-methylglutaryl-CoA synthase, Hypoglycemia, Ketogenesis

## Abstract

**Background:**

Mitochondrial 3-hydroxy-3-methylglutaryl-CoA synthase (mHS) deficiency is an autosomal recessive inborn error of metabolism, which will give rise to failure of ketogenesis in liver during illness or fasting. It is a very rare disease with only a few patients reported worldwide, most of which had a good prognosis after proper therapies.

**Case presentation:**

We report a 9-month-old boy with mHS deficiency presenting with unusually severe and persistent acidosis after diarrhea and reduced oral food intake. The metabolic acidosis persisted even after supplementation with sugar and alkaline solution. Blood purification and assisted respiration alleviated symptoms, but a second onset induced by respiratory infection several days later led to multiple organ failure and death. Urine organic acid analysis during the acute episode revealed a complex pattern of ketogenic dicarboxylic and 3-hydroxydicarboxylic aciduria with prominent elevation of glutaric acid and adipic acid, which seem to be specific to mHS deficiency. Plasma acylcarnitine analysis revealed elevated 3-hydroxybutyrylcarnitine and acetylcarnitine. This is the first report of elevated 3-hydroxybutyrylcarnitine in mHS deficiency. Whole exome sequencing revealed a novel compound heterozygous mutation in *HMGCS2* (c.100C > T and c.1465delA).

**Conclusion:**

This severe case suggests the need for patients with mHS deficiency to avoid recurrent illness because it can induce severe metabolic crisis, possibly leading to death. Such patients may also require special treatment, such as blood purification. Urine organic acid profile during the acute episode may give a hint to the disease.

## Background

Mitochondrial 3-hydroxy-3-methylglutaryl-CoA (HMG-CoA) synthase deficiency (mHS deficiency) is an autosomal recessive disorder in which hepatic ketogenesis fails during illness or fasting [1]. At least two isoforms of HMG-CoA synthase are expressed in the body, one in the cytoplasm and one in the mitochondrial matrix [2]. Mitochondrial HMG-CoA synthase catalyzes the condensation of acetyl-CoA and acetoacetyl-CoA to form HMG-CoA in the first step of ketogenesis which breaks down fatty acids to provide energy during carbohydrate deprivation.

mHS deficiency is a rare disorder, with fewer than 20 patients reported worldwide [3, 4]. The first case was reported in 1997 and involved a 6-year-old boy of Chinese descent who experienced, after mild gastroenteritis, a brief generalized seizure and hypoglycemia with negative ketones, which left him semi-comatose [5]. Subsequent case reports involved symptoms including diarrhea, hepatomegaly, lethargy and hypoglycemia, which improved after glucose infusion [6, 7]. Acidosis was also reported in some cases, and general prognosis was good [8, 9]. The diagnosis of mHS deficiency is very difficult because patients are generally asymptomatic unless exposed to starvation or infection [10]. Measurement of HMG-CoA synthase activity and urine organic acids spectrum during symptomatic episodes can hint at deficiency of this enzyme, but a confirmation of diagnosis can be obtained only by genetic testing.

Here, we describe a 9-month-old Chinese infant with two novel heterozygotic variants (c.100C > T and c.1465delA) in the *HMGCS2* gene. The patient displayed unusually severe clinical symptoms including hypoglycemia, Kussmaul breathing, and persistent and intractable metabolic acidosis after diarrhea. These symptoms were improved by a course of blood purification, but he had a very poor prognosis and died after a second onset of disease. This severe case contrasts with previous patients with mHS deficiency, who had relatively good prognosis.

## Case presentation

The patent was a 9-month-old Chinese boy who was the second child at full term with no abnormal results during newborn screening. He had an 18-year-old brother who is healthy so far and his parents are also healthy. However, two of the mother’s siblings were dead with unknown reasons.

The boy presented at a local hospital with diarrhea that had persisted during the previous 10 days. The boy had previously been diagnosed with sepsis, infectious dermatitis, and myocardial injury during the neonatal period in a local hospital, and he had recovered after 10 days of treatment. He had no other remarkable history.

**Fig. 1:**
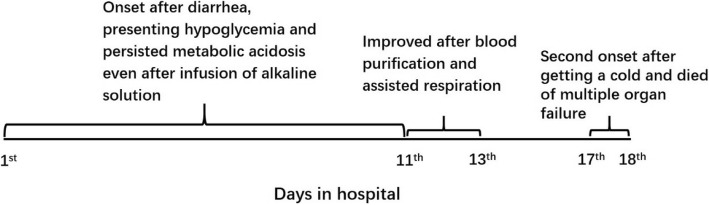
The clinical course of the patient during disease process in the hospital

Blood-gas analysis at the local hospital showed a glucose concentration of 0.7 mmol/L (normal, 3.3–5.3 mmol/L), with a pH of 7.20 (7.35–7.45), P_CO2_ of 13.8 mmHg (35–45 mmHg), P_O2_ of 56.7 mmHg (80–100 mmHg), HCO_3_^−^ concentration of 5.3 mmol/L (21.4–27.3 mmol/L), and actual base excess of − 20.8 mmol/L (− 3 to 3 mmol/L). His condition continued to deteriorate despite treatment, so he was sent to our hospital. At admission to our center, the boy was languid that suggested worsening of disease in the absence of obvious causes. His milk was reduced to half the normal amount, and intravenous glucose was administered to treat hypoglycemia. However, his symptoms did not improve and he became dysphoric at night and had difficulty sleeping. His response rates were low, and he developed polypnea and cyanosis of the face and lips with groaning. Blood-gas analysis revealed a glucose concentration of 4.3 mmol/L (3.3–5.3 mmol/L), with a pH of 7.3 (7.35–7.45), HCO_3_^−^ concentration of 5.4 mmol/L (21.4–27.3 mmol/L), total CO_2_ of 5.7 mmol/L (24–32 mmol/L), actual base excess of − 21 mmol/L (− 3 to 3 mmol/L), standard base excess of − 18 mmol/L (− 3 to 3 mmol/L), standard bicarbonate of 10.9 mmol/L (21.3–24.8 mmol/L), alanine aminotransferase (ALT) levels of 124 U/L (0–55 U/L), and aspartate transaminase (AST) levels of 339 U/L (0–55 U/L). His red blood cell count was 3.48 × 10^12^/L (4.0–5.3) with a hemoglobin concentration of 84 g/L (120–160 g/L), and he had a mean corpuscular volume of 77.6 fl (80–100 fl), mean corpuscular hemoglobin of 24.1 pg (26–32 pg), and mean corpuscular hemoglobin concentration of 311 g/L (320–360 g/L). Magnetic resonance imaging revealed slight widening of the sulci and the frontal and temporal lobe clefts in both cerebral hemispheres, without prominent abnormalities in the cerebral parenchyma. Metabolic acidosis was recurrent, severe, and worsened even after infusion of alkaline solution. The patient developed progressive dyspnea, Kussmaul breathing, and the three concave signs. Blood purification and assisted respiration were initiated, which gradually alleviated metabolic acidosis, and the patient was discharged. Unfortunately, he contracted a cold and cough 2 days after leaving hospital and the same symptoms reappeared in more severe form. The boy died of multiple organ failure soon after re-admission (Fig. 1).

**Fig. 2:**
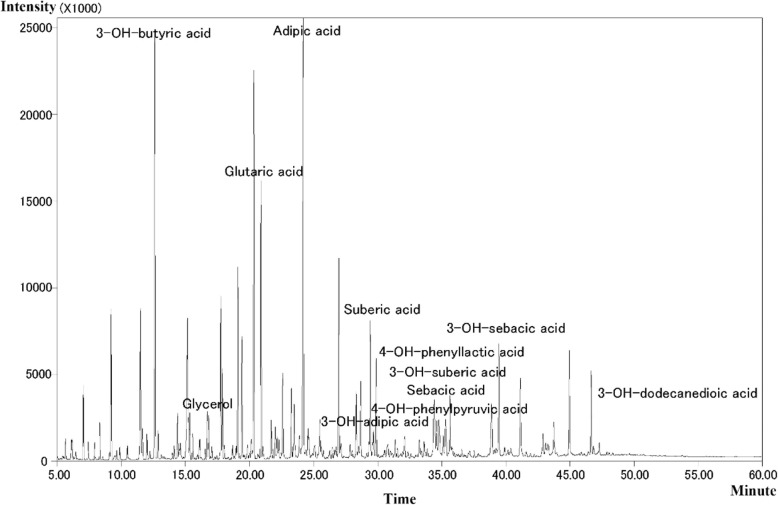
Chromatogram of urine organic acids from our patient. Urine was sampled during an acute episode of metabolic disturbance involving acidosis. The results indicate significant elevation of 3-hydroxybutyric acid, glutaric acid, adipic acid glycerol; as well as moderate elevation of dicarboxylic acids, 3-hydroxydicarboxylic acids, 4-hydroxyphenyllactate acids

Urine organic acid analysis were performed during an acute episode of metabolic disturbance involving acidosis after the patient’s first admission to our hospital. A complex pattern of metabolites was observed (Fig. 2, Table 1). This pattern showed highly elevated levels of the following metabolites, quantified as ratios of the metabolite peak to internal standard peak on gas chromatographs: 3-hydroxybutyric acid (391.21, reference: 0–3.7), acetoacetic acid (4.26, reference: 0–0), glutaric acid (220.52, reference: 0.0–4.0), adipic acid (195.82, reference: 0.0–5.0), and glycerol (39.26, reference: 0.0–0.8); moderately elevated dicarboxylic and 3-hydroxydicarboxylic acids such as ethylmalonic acid (11.35, reference: 0.0–6.2), suberic acid (37.34, reference: 0.3–4.7), sebacic acid (18.73, reference: 0.4–7.0), 3-hydroxysuberic acid (30.66, reference: 0.0–4.8), 3-hydroxysebacic acid (28.47, reference: 0.0–4.4), and 3-hydroyxdodecanedioic acid (14.70, reference: 0.0–1.4). Our patient also showed slightly elevated 4-hydroxyphenyllactic acid (77.23, reference: 0.0–7.0) and 4-hydroxyphenylpyruvic acid (2.10, reference: 0.0–0.9). Plasma acylcarnitine analysis revealed highly elevated 3-hydroxybutyrylcarnitine (1.45 μmol/L, reference: 0.05–0.44 μmol/L) and acetylcarnitine (75.37 μmol/L, reference: 5.5–36 μmol/L), as well as slightly elevated butyrylcarnitine (0.65 μmol/L, reference: 0.06–0.5 μmol/L).

**Table 1:** Urine organic acid profiles and genetic mutations reported for individuals with HMG-CoA synthase deficiency

Study	Plasma acylcarnitines	Urinary organic acids	*HMGCS2* mutation	Prognosis
Thompson et al. (1997) [5]	Normal	Normal	Exon 2, 520 T > C, homozygous	Improved
Morris et al. (1998) [12]	Normal	Dicarboxylic aciduria	Exon 7, c.1270C > T, homozygous	Improved
Aledo et al. (2001) [13]	Normal	Dicarboxylic aciduria without adequate ketonuria	Exon 3, c.634G > A Exon 9, c.1499G > A	Improved
Zschocke et al. (2002) [7]	Normal	nonspecific	Exon 3, c.634G > A Intron 5, IVS5 + 1 g > a	Improved
Wolf et al. (2003) [8]	Normal	Dicarboxylic aciduria	Exon 2, c.160G > A Exon 2, c.500A > G	Improved
Aledo et al. (2006) [9]	C0↓, C2↑	Dicarboxylic aciduria	c.614G > A c.971 T > C	Improved
Ramos et al. (2013) [14]	Normal	Ketonuria with dicarboxylic aciduria	Exon 6, c.1162G > A, Exon 7, c.1270C > T	Improved
Conboy et al. (2017) [3]	C2↑↑↑	Glutaric acid↑↑↑, adipic acid ↑↑↑, 4-hydroxyphenyl lactate↑, 4-hydroxyphenyl pyruvate↑	c.409A > T c.1141A > G	Improved, but profound developmental delay
Ma Dan, Yu Dan (2018) [4]	Not mentioned	Glutaric acid↑↑↑, dicarboxylic aciduria	Exon 9, c.1502G > A, homozygous	Improved
Present report	C2↑↑↑, C4↑, C4-OH↑↑↑	3-hydroxybutyric acid↑↑↑, glutaric acid↑↑↑, adipic acid ↑↑↑, glycerol↑↑↑, 4-hydroxyphenyllactate↑↑, 4-hydroxyphenyl pyruvate↑	Exon 1, c.100C > T Exon 9, c.1465del A	Death

↑↑↑ = highly elevated, ↑↑ = moderately elevated, ↑ = slightly elevated

C0 = free carnitine, C2 = acetylcarnitine, C4 = butyrylcarnitine, C4-OH = 3-hydroxybutyrylcarnitine

**Fig. 3:**
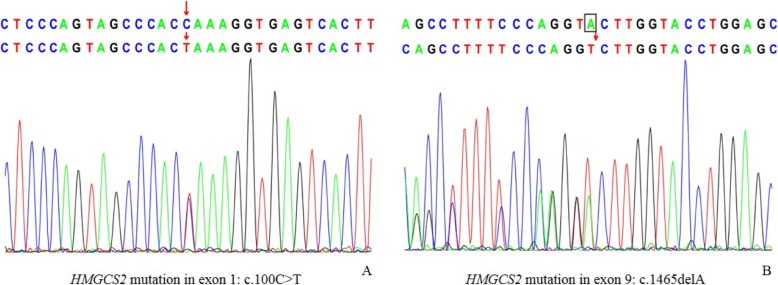
The spectrum of *HMGCS2* mutations in the patient. **a**: c.100C > A was identified in exon 1 on chromosome 1. **b**: c:.1464delA was identified in exon 9 on chromosome 1

Strongly suspecting an inborn error of metabolism, so we performed whole-exome sequencing and compared the results against a metabolic disorder panel. Sequencing revealed a compound heterozygous mutation in *HMGCS2* involving a paternally inherited c.100C > T substitution in exon 1, which resulted in premature translation termination (p.Q34X, 475); and a maternally inherited c.1465delA mutation in exon 9, which resulted in a frameshift and premature translation termination (p.T489Lfs*55) (Fig. 3). Both variants were classified as pathogenic according to ACMG variant classification guidelines [11]. Moreover, some single heterozygous mutations were also identified such as ALDOB, DGKE and LRBA. However, these diseases caused by the mutations are autosomal recessive inheritance which means a single variant will not cause the disease theoretically.

## Discussion and conclusions

mHS deficiency is a rare inborn error of metabolism that typically manifests in the first year of life after prolonged fasting or illness. In this study, we describe a patient with this deficiency who presented with unusually severe acidosis associated with a novel compound heterozygous mutation in *HMGCS2* at two loci (c.100C > T and c.1465delA), both of which result in premature termination of the HMG-CoA synthase.

Previous studies of patients with mHS deficiency reported a good prognosis and alleviation of metabolic acidosis after intravenous glucose infusion (Table 1), with exception of one patient who showed profoundly delayed growth and development [3]. In contrast, the patient in our study had more severe symptoms, including persistent and severe metabolic acidosis that was not corrected by glucose and bicarbonate infusion and improved only after blood purification and assisted respiration. This observation is similar to a previous study of a patient with mHS deficiency, whose severe metabolic acidosis could be corrected only by renal replacement therapy [4]. These findings indicate that patients with this deficiency can vary widely in their disease severity and response to treatment. Subsequent infection in our patient induced acute metabolic crisis, followed rapidly by deep coma and multiple organ failure resulting in death. We propose that precautions should be taken to avoid repeated illness in patients with mHS deficiency, especially those with a severe manifestation of disease, because such illness can precipitate more severe outcomes. Our patient also exhibited microcytic hypochromic anemia, which has not previously been observed in patients with mHS deficiency. This may be linked to the especially severe symptoms observed and should be further investigated.

Profiling of urine organic acids during an acute episode of metabolic disturbance involving acidosis revealed an unusual pattern of dicarboxylic aciduria and 3-hydroxydicarboxylic aciduria, with prominent elevation of glutaric acid and adipic acid. This metabolite profile is similar to that described in other patients with mHS deficiency [3, 4] (Table 1). Elevation of dicarboxylic, and 3-hydroxy dicarboxylic acids is also commonly seen in glutaric aciduria type II (GA-II), where the body is unable to completely break down branched-chain amino acids and fatty acids due to a deficiency in multiple acyl-CoA dehydrogenases [12, 15]. However, we considered GA-II to be unlikely when we found there were no multiple acyl-carnitines elevated in plasma which is specific for GA-II. Dicarboxylic and 3-hydroxy dicarboxylic acids are thought to be produced by omega-oxidation when beta-oxidation of fatty acids is inhibited due to inefficient ketogenesis [13, 16]. Although 3-hydroxybutyric acid is not usually detectable in this disease (Table 1), we detected it in our patient, and in fact the levels were even higher than in a previously described patient with detectable levels [3]. The detection of 3-hydroxybutyric acid in these two patients suggests that ketones can be synthesized via metabolic pathways involving the breakdown of ketogenic amino acids or an increase in the amount of l-isomer, which is produced in the final steps of fatty acid oxidation, relative to the amount of d-isomer, which is observed in fasting ketosis [3]. Hypoglycemia with ketone elevation does not exclude a diagnosis of mHS deficiency. We also observed a prominent increase in glycerol as well as a slight increase in 4-hydroxyphenyllactate and 4-hydroxyphenyl pyruvate. Disorders of fatty acid oxidation typically involve elevated dicarboxylic acid and 3-hydroxydicarboxylic acid, but not elevated glycerol. Elevation of 4-hydroxyphenyllactate and 4-hydroxyphenyl pyruvate can also indicate hepatic injury.

The spectrum of elevated acylcarnitines in our patient showed highly elevated 3-hydroxybutyrylcarnitine and acetylcarnitine with slightly elevated butyrylcarnitine, which has not been seen in previous reports of mHS deficiency (Table 1). Elevation of acetylcarnitine in patients with this disease has been attributed to l-carnitine supplementation, and may indicate increased production of acetyl-CoA, the final product in fat oxidation [3]. Increased 3-hydroxybutyrylcarnitine is also commonly seen in congenital hyperinsulinism, 3-hydroxyacyl-coenzyme dehydrogenase deficiency, or patients with beta-ketothiolase deficiency [14, 17]. The metabolite 3-hydroxybutyrylcarnitine is located primarily in the cytoplasm, and can be synthesized from 3-hydroxybutyric acid. Increased 3-hydroxybutyrylcarnitine could therefore be due to the large amount of 3-hydroxybutyric acid [15, 18]. However, it is not a specific or reliable marker for mHS deficiency.

In this report, we describe a severe case of mHS deficiency involving a novel compound heterozygous mutation in *HMGCS2*. A diagnosis of mHS deficiency should be considered when a patient presents with hypoglycemia and metabolic acidosis after fasting or diarrhea, accompanied by highly elevated glutaric, adipic with other dicarboxylic, and 3-hydroxydicarboxylic acids. In contrast to previous reports, our case highlights the potential severity of HMG-CoA synthase deficiency and suggests that serious cases should be protected from recurrent illness.

## Data Availability

All data used analyzed during the current study are included in this published article.

## Abbreviations

ALT: Alanine aminotransferase
AST: Aspartate transaminase
C0: Free carnitine
C2: Acetylcarnitine
C4: Butyrylcarnitine
C4-OH: 3-hydroxybutyrylcarnitine
HMG-CoA: 3-hydroxy-3-methylglutaryl-CoA
mHS: mitochondrial 3-hydroxy-3-methylglutaryl-CoA synthase
